# A c-Src Inhibitor Peptide Based on Connexin43 Exerts Neuroprotective Effects through the Inhibition of Glial Hemichannel Activity

**DOI:** 10.3389/fnmol.2017.00418

**Published:** 2017-12-15

**Authors:** Ester Gangoso, Rocío Talaverón, Myriam Jaraíz-Rodríguez, Marta Domínguez-Prieto, Pascal Ezan, Annette Koulakoff, José M. Medina, Christian Giaume, Arantxa Tabernero

**Affiliations:** ^1^MEMOLIFE Laboratory of Excellence and Paris Science Lettre Research University, Center for Interdisciplinary Research in Biology (CIRB)/Centre National de la Recherche Scientifique, Unité Mixte de Recherche 7241/Institut National de la Santé et de la Recherche Médicale U1050, Collège de France, Université Pierre et Marie Curie, Paris, France; ^2^Departamento de Bioquímica y Biología Molecular, Instituto de Neurociencias de Castilla y León (INCYL), Universidad de Salamanca, Salamanca, Spain

**Keywords:** connexin, c-Src, hemichannels, excitotoxicity, neuroinflammation, neuroprotection, neurons, astrocytes

## Abstract

The non-receptor tyrosine kinase c-Src is an important mediator in several signaling pathways related to neuroinflammation. Our previous study showed that cortical injection of kainic acid (KA) promoted a transient increase in c-Src activity in reactive astrocytes surrounding the neuronal lesion. As a cell-penetrating peptide based on connexin43 (Cx43), specifically TAT-Cx43_266–283_, inhibits Src activity, we investigated the effect of TAT-Cx43_266–283_ on neuronal death promoted by cortical KA injections in adult mice. As expected, KA promoted neuronal death, estimated by the reduction in NeuN-positive cells and reactive gliosis, characterized by the increase in glial fibrillary acidic protein (GFAP) expression. Interestingly, TAT-Cx43_266–283_ injected with KA diminished neuronal death and reactive gliosis compared to KA or KA+TAT injections. In order to gain insight into the neuroprotective mechanism, we used *in vitro* models. In primary cultured neurons, TAT-Cx43_266–283_ did not prevent neuronal death promoted by KA, but when neurons were grown on top of astrocytes, TAT-Cx43_266–283_ prevented neuronal death promoted by KA. These observations demonstrate the participation of astrocytes in the neuroprotective effect of TAT-Cx43_266–283_. Furthermore, the neuroprotective effect was also present in non-contact co-cultures, suggesting the contribution of soluble factors released by astrocytes. As glial hemichannel activity is associated with the release of several factors, such as ATP and glutamate, that cause neuronal death, we explored the participation of these channels on the neuroprotective effect of TAT-Cx43_266–283._ Our results confirmed that inhibitors of ATP and NMDA receptors prevented neuronal death in co-cultures treated with KA, suggesting the participation of astrocyte hemichannels in neurotoxicity. Furthermore, TAT-Cx43_266–283_ reduced hemichannel activity promoted by KA in neuron-astrocyte co-cultures as assessed by ethidium bromide (EtBr) uptake assay. In fact, TAT-Cx43_266–283_ and dasatinib, a potent c-Src inhibitor, strongly reduced the activation of astrocyte hemichannels. In conclusion, our results suggest that TAT-Cx43_266–283_ exerts a neuroprotective effect through the reduction of hemichannel activity likely mediated by c-Src in astrocytes. These data unveil a new role of c-Src in the regulation of Cx43-hemichannel activity that could be part of the mechanism by which astroglial c-Src participates in neuroinflammation.

## Introduction

Neuronal excitotoxicity mediated by glutamate receptors contributes to a variety of disorders in the central nervous system (CNS), including acute insults like ischemic stroke or neurodegenerative diseases, such as Alzheimer’s disease, Parkinson’s disease or Huntington’s chorea (Zhang and Zhu, 2011; Lewerenz and Maher, 2015). The initial neuronal excitotoxic damage causes reactive gliosis, an inflammatory response characterized by the proliferation and activation of microglia and astrocytes, which contributes to the outcome of the neurodegenerative process. Excitotoxic neuronal death is commonly induced experimentally in rodents by the administration of kainic acid (KA), a potent agonist to the AMPA/kainate class of glutamate receptors.

Our previous studies showed that after a cortical lesion induced by a KA injection, astrocytes within the area depleted in neurons reacted with an increase in glial fibrillary acidic protein (GFAP) and a decrease in connexin43 (Cx43) expression (Koulakoff et al., 2008), the main protein that forms gap junction channels and hemichannels in astrocytes (Giaume et al., 2010, 2013). Furthermore, we found a transient increase in c-Src activity in this region surrounding the neuronal lesion (Gangoso et al., 2012).

c-Src, is a non-receptor tyrosine kinase very well-known because of its oncogenic properties in many cancers. However, c-Src also plays an important role in inflammatory signaling (Byeon et al., 2012; Liu et al., 2014). Within the CNS, the participation of c-Src signaling in neuroinflammation has been well documented (Song et al., 2016), for instance mice lacking c-Src show decreased infarct volumes after stroke (Paul et al., 2001). This study elegantly demonstrates that c-Src represents a key intermediate in the pathophysiology of cerebral ischemia, where it appears to regulate neuronal damage by influencing vascular endothelial growth factor-mediated vascular permeability. Similarly, the inhibition of c-Src, through the reduction of neuroinflammation, promotes a neuroprotective effect in Parkinson’s disease and Alzheimer’s disease models (Dhawan and Combs, 2012; Tai et al., 2013).

We have recently shown that Cx43, through the interaction with c-Src and its endogenous inhibitors c-terminal Src kinase (CSK) and phosphatase and tensin homolog (PTEN), inhibits c-Src activity in astrocytes and glioma cells (Herrero-González et al., 2010; González-Sánchez et al., 2016; Tabernero et al., 2016). In fact, a cell-penetrating peptide based on this interacting region, TAT-Cx43_266–283_, is sufficient to recruit c-Src, CSK and PTEN and to inhibit c-Src activity in different types of glioma cells (Gangoso et al., 2014; González-Sánchez et al., 2016; Jaraíz-Rodríguez et al., 2017). As c-Src activity, an important mediator in neuroinflammation, is increased after an excitotoxic neuronal lesion (Gangoso et al., 2012), we presently investigated the effect of TAT-Cx43_266–283_ on neuronal death promoted by KA.

## Materials and Methods

### Animals

C57BL6 mice were maintained in the animal facility of the Collège de France (Paris). Animal experimentation was carried out in accordance with the European Community Council Directives (2010/63/UE) and care was taken to minimize their suffering. The study with C57BL6 mice was approved by the bioethics committee of the Collège de France (Paris). Albino Wistar rats were obtained from the animal house of the University of Salamanca (Spain) and were used according to local and EU Ethical Committee guidelines. The study with Albino Wistar rats was approved by the bioethics committee of the University of Salamanca and Junta de Castilla y León (Spain).

### Peptides

Synthetic peptides (>85% pure) were obtained from GenScript (Piscataway, NJ, USA). YGRKKRRQRRR was used as the TAT sequence, which is responsible for the cell penetration of the peptides. The sequence of TAT-Cx43_266–283_ was TAT-AYFNGCSSPTAPLSPMSP (Gangoso et al., 2014).

### Kainic Acid Lesion and Peptide Administration in Mice

Adult mice were subjected to intracerebral injection as previously described (Koulakoff et al., 2008; Gangoso et al., 2012). Briefly, mice were deeply anesthetized by intraperitoneal injection of 0.3 mL of 2% avertin and were then subjected to a low-pressure injection of 1 nmol KA (Sigma-Aldrich, St. Louis, MO, USA) in 1 μL phosphate-buffered saline (PBS) in controls, 1 nmol KA plus 1 nmol TAT in 1 μl PBS, or 1 nmol KA plus 1 nmol TAT-Cx43_266–283_ in 1 μl PBS under stereotaxic guidance (coordinates: −2.1 mm anteroposterior, 1.5 mm mediolateral and 0.6 mm dorsoventral from Bregma) aiming the injection into the right cerebral cortex. Animals were sacrificed by cervical dislocation at 7 days post-injection.

### Quantification of the Extent of Neuronal Lesions in Mice

Cryostat sections (20 μm) prepared as previously described (Mei et al., 2010) were fixed for 30 min at room temperature with 4% paraformaldehyde, washed in PBS and pre-incubated for 1 h in PBS containing 0.2% gelatine and 0.2% Triton-X100. Brain sections were incubated overnight at 4°C with rabbit polyclonal antibody anti-GFAP (1:1000, G9269; Sigma) and mouse monoclonal anti-NeuN (1:500, MAB377; Merck Millipore, Madrid, Spain). After three washes with PBS, sections were incubated for 2 h at room temperature with their corresponding Alexa Fluor secondary antibodies (1:2000; Life Technologies, Carlsbad, CA, USA). After several washes, slices were mounted in Fluoromount (Southern Biotechnologies, Birmingham, AL, USA) and examined with an epifluorescence microscope (Eclipse E800; Nikon, Tokyo, Japan).

**Figure 1:**
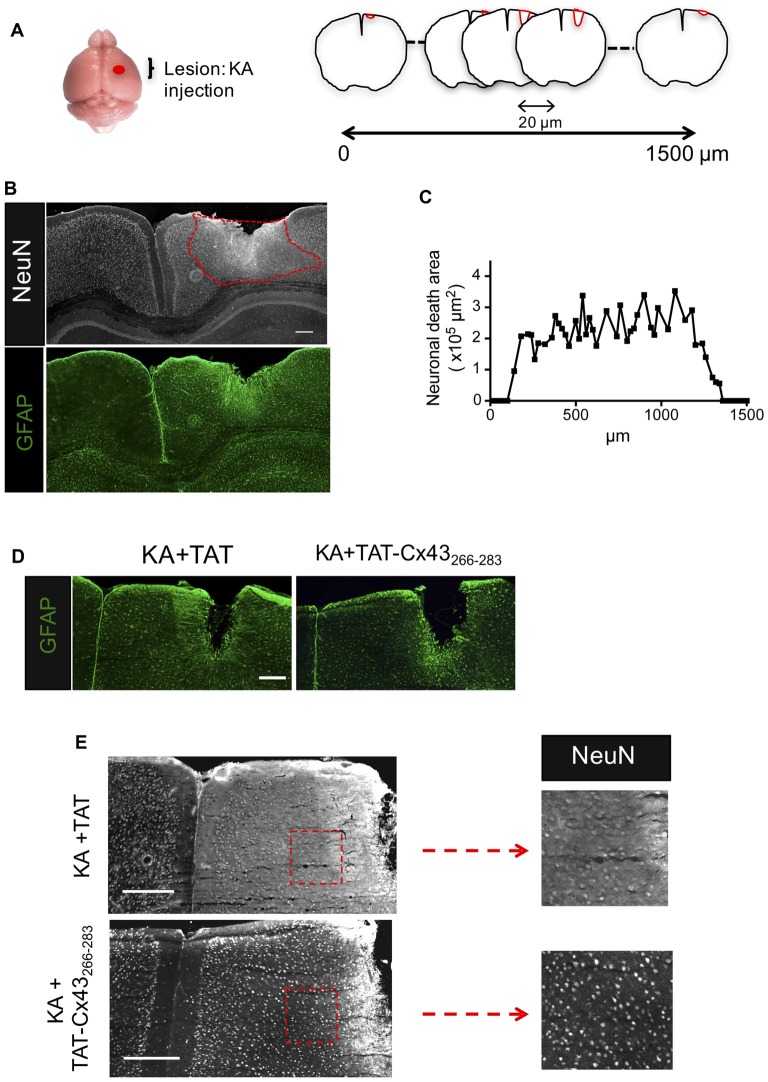
Effect of TAT-connexin43 (Cx43)_266–283_ on NeuN and glial fibrillary acidic protein (GFAP) expression in adult mouse brain after kainic acid (KA) injection.** (A)** Schematic of the experimental strategy used to analyze neuronal injury promoted by 1 nmol/μl KA injection. **(B)** Expression of NeuN and GFAP in the cortex, 7 days after KA injection. Representative photomicrographs from the same field at the level of the lesioned area delimited by the dashed red line. **(C)** Quantification of neuronal death area along the rostrocaudal axis. Representative photomicrographs of GFAP **(D)** and NeuN **(E)** immunohistochemical staining in brain sections at the level of the lesioned area, 7 days after the injection of 1 nmol/μl KA + 1 nmol/μl TAT or 1 nmol/μl KA + 1 nmol/μl TAT-Cx43_266–283_. Higher magnification photomicrographs showing the decrease in NeuN-positive cells after the injection of KA+TAT compared to KA+TAT-Cx43_266–283_. Bar, 250 μm.

For quantification, the whole area of the lesion was analyzed using Fiji (Schindelin et al., 2012). Every 20 μm across the entire lesion (1500 μm), the damaged area was drawn in each slice with the freehand selection tool (Figure 1A). For drawing, we followed as criteria a reduction in NeuN staining within neurons compared with the contralateral region. The necrotic tissue was included as part of the lesion (Figure 1B). Sometimes the necrotic tissue was lost but that area was counted, assuming it was damaged tissue. Although some slices where lost during the sectioning process, all the conditions analyzed comprised at least 80% of the lesion. The areas across the whole lesion were plotted against their location using Prism software. Cavalieri’s principle was used to estimate the volume of the lesion (Jelsing et al., 2005). Briefly, the volume was estimated multiplying the summatory of the areas, obtained as described previously, by the distance between the sections analyzed along the rostro-caudal axis.

### Neuron and Astrocyte Cultures

Primary cultures were obtained as previously described (Tabernero et al., 1993). For neuron cultures, rat fetuses at 17.5 days of gestation were delivered by rapid hysterectomy after cervical dislocation of the mother. Postnatal 1-day newborn rats were used to prepare astrocytes in culture. Briefly, animals were decapitated and their brains immediately excised. After removing the meninges and blood vessels, the forebrains were placed in Earle’s balanced solution (EBS) containing 20 μg/ml DNase and 0.3% (w/v) BSA. The tissue was minced, washed, centrifuged and incubated in 0.025% trypsin (type III) and 60 μg/ml DNase I for 15 min at 37°C. Trypsinization was terminated by the addition of Dulbecco’s Modified Eagle’s Medium (DMEM) containing 10% fetal bovine serum (FCS; Gibco, Life Technologies). The tissue was then dissociated by gently passing it eight times through a siliconized Pasteur pipette, and the supernatant cell suspension was recovered. This operation was repeated and the resulting cell suspension was centrifuged. The cells were then resuspended in DMEM containing 10% FCS and plated on Petri dishes coated with 10 μg/ml poly-L-lysine at a density of 10^5^ cells/cm^2^. Cells were maintained at 37°C and 5% CO_2_. One day after plating, 10 μM cytosine arabinoside was added to avoid glial cell proliferation on neuronal cultures.

### Neuron-Astrocyte Co-cultures

For neuron-astrocyte co-cultures, the cell suspension obtained for neuron culture was plated at a density of 3.75 × 10^4^ cells/cm^2^ on confluent 21 days *in vitro* (DIV) astrocytes. These co-cultures were maintained at 37°C and 5% CO_2_ in DMEM + 10% FCS for 7 days and then different treatments were applied for 8 h.

For non-contact neuron-astrocyte co-cultures, the cell suspension obtained for neuron culture was plated at a density of 10^5^ cells/cm^2^ in 12-well plates coated with 10 μg/ml poly-L-lysine. Cells were maintained at 37°C and 5% CO_2_ and 1 day after plating, cytosine arabinoside was added to avoid glial cell proliferation. Eighteen DIV astrocytes were plated in 500 μL DMEM + 10% FCS on inserts containing polyethylene terephthalate filters with 1-μm pores (Merck Millipore) at 10^5^ cells/cm^2^, whereas 1 ml DMEM + 10% FCS was added to the lower well. After 3 days, the medium of the astrocytes was changed and the inserts were placed on top of 4 DIV neurons with 25% of the medium changed. These non-contact co-cultures were maintained at 37°C and 5% CO_2_ in DMEM + 10% FCS in the presence of the different treatments for 3 days.

### Cell Treatments

All treatments were added to the culture medium and maintained at 37°C for the indicated times. The treatments were as follows: 50 μM TAT, 50 μM TAT-Cx43_266–283_, 100 μM KA, 10 μg/ml lipopolysaccharide (LPS; Sigma), 200 μM carbenoxolone (CBX; hemichannel inhibitor, Sigma), 1 μM dasatinib (c-Src inhibitor; Selleck Chemicals, Munich, Germany), dimethyl sulfoxide (vehicle for dasatinib; 1 μl/ml), 20 μM 3-(2-carboxypiperazin-4-yl)propyl-1-phosphonic acid (CPP; NMDA receptor blocker), 200 μM Adenosine 5′-triphosphate, periodate oxidized sodium salt (oATP; P2X receptor blocker, Sigma) and 100 μM Brilliant Blue G (BBG; P2X7 receptor blocker, Sigma).

### Immunocytochemistry

Cells were fixed with 4% (w/v) paraformaldehyde in PBS for 20 min and blocked for 30 min in antibody diluting solution (PBS containing 10% FCS, 0.1 M lysine and 0.02% sodium azide). Cells were then incubated overnight at 4°C with mouse anti-NeuN (1:100) and for 2 h with the secondary antibody anti-mouse labeled with Alexa Fluor 488 (A11029; Life Technologies) all prepared in antibody diluting solution containing 0.1% Triton-X100. Nuclei were stained with 4′,6′-diamidino-2-phenylindole (DAPI; 1.25 μg/ml; Invitrogen) for 10 min. Cells were then mounted using the Slowfade Gold Antifade Kit (ThermoFisher) and analyzed on a Nikon inverted fluorescence microscope connected to a digital video camera (DC100; Leica, Wetzlar, Germany). Negative controls carried out by omission of the primary antibodies resulted in absence of staining in all cases. At least six photomicrographs were taken from each plate. The number of nuclei (DAPI staining) and NeuN-positive cells were counted with ImageJ (NIH, Bethesda, MD, USA) on 8-bit images. The percentage of NeuN-positive cells was calculated from the total number of cells (DAPI staining).

### MTT Assay

Cells cultured at 37°C were incubated in the dark for 75 min with culture medium containing 0.5 mg/ml MTT (Sigma). The medium was then removed, and the cells were incubated for 10 min in the dark with dimethyl sulfoxide with mild shaking. Finally, the absorbance was measured at a wavelength of 570 nm using a microplate reader (Appliskan 2001; Thermo Electron Corporation, Thermo Scientific, Madrid, Spain).

### Ethidium Bromide Uptake Analyses in Cell Cultures

Cultured cells were incubated with 5 μM ethidium bromide (EtBr) in HEPES-buffered salt solution (140 mM NaCl, 5.4 mM KCl, 1.8 mM CaCl_2_, 1 mM MgCl_2_, 10 mM glucose and 5 mM HEPES; pH 7.4) for 10 min. Cells were then washed with the same buffer, fixed with 4% paraformaldehyde in PBS and analyzed on a Nikon inverted fluorescence microscope connected to a digital video camera (Leica DC100). The EtBr fluorescence intensity within the nucleus was measured with the ImageJ software and the average of data from six images from different fields in the same culture was calculated to obtain the final measurement of dye uptake in each culture.

### Assessment of Gap Junctional Intercellular Communication

Gap junction permeability was determined by the scrape-loading/dye transfer technique as previously described (Herrero-González et al., 2010). Scrape-loading was performed by scraping the cell layer with a broken razor blade in a Ca^2+^-free ionic solution containing Lucifer yellow (LY; 1 mg/ml). LY is a low molecular weight (457 Da) fluorescent dye that can pass through the gap junctions of loaded cells to their neighbors. After 2 min, the dye solution was removed and the cells were carefully washed. Subsequently, 8 min after scraping, fluorescence photomicrographs were captured with a digital video camera (Leica DC100) connected to an inverted fluorescent microscope equipped with the appropriate filters (Diaphot, Nikon). At least six photomicrographs of the center of the dish were taken and the number of fluorescent cells per field were counted.

### Statistical Analysis

Results are expressed as the mean ± SEM of at least three independent experiments. Statistical analyses were carried out in Sigma Plot 11 (Systat Software) using an analysis of variance (one-way ANOVA), followed by the Holm-Sidak method for multiple comparisons at a significant level of *p* < 0.05.

## Results

### TAT-Cx43_266–283_ Exerts Neuroprotective Effects in *in Vivo* KA-Induced Cortical Lesions

Following the method previously described (Koulakoff et al., 2008), KA was cortically injected and after 7 days, brains were dissected, fixed and 20-μm frontal sections were cut along 1500 μm to cover the entire lesion (Figure 1A). Neuronal death was estimated by measuring the area in which neurons contain a reduced NeuN intensity when compared to contralateral region (Figure 1B), as described in “Materials and Methods” section. In agreement with previous results (Koulakoff et al., 2008), KA promoted neuronal death and reactive gliosis, characterized by the increase in GFAP expression (Figure 1B). Figure 1C shows neuronal death areas along the rostrocaudal axis found 7 days after KA injection.

Next, we analyzed the effect of TAT-Cx43_266–283_ on neuronal death promoted by KA using TAT penetrating peptide as a control. TAT-Cx43_266–283_ was injected with KA and its effect was compared with KA or KA+TAT. Our results showed that TAT-Cx43_266–283_ diminished reactive gliosis as judged by GFAP staining (Figure 1D). More importantly, TAT-Cx43_266–283_ prevented the reduction in NeuN-positive cells promoted by KA (Figure 1E).

**Figure 2:**
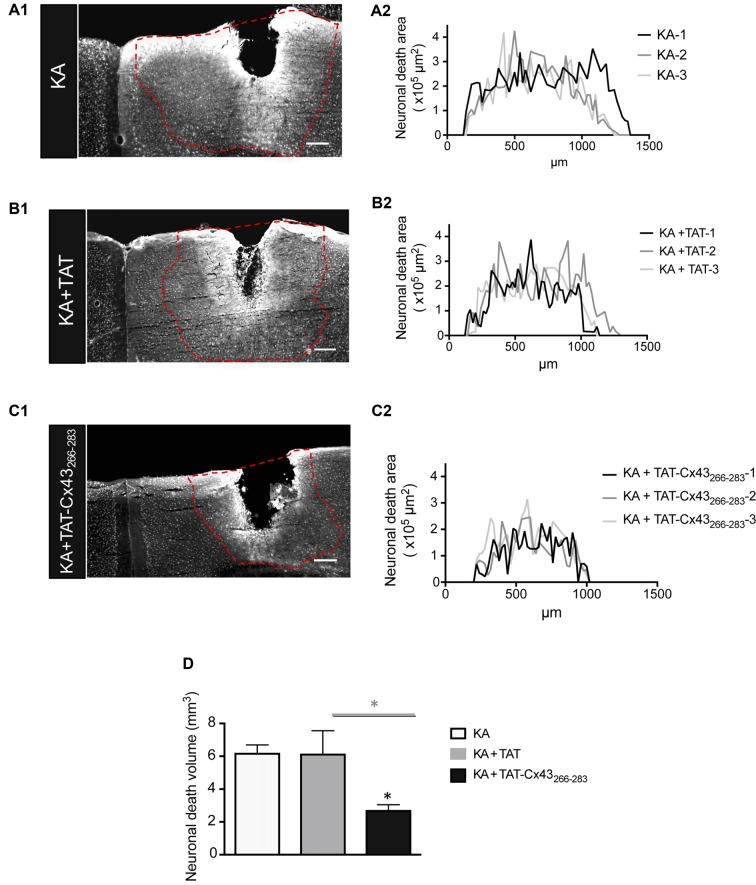
Effect of TAT-Cx43_266–283_ on the extent of neuronal death in adult mouse brain cortex after KA injection. Representative photomicrographs of NeuN immunohistochemical staining in brain sections at the level of the lesioned area, 7 days after 1 nmol/μl KA injection **(A1)**, KA+TAT **(B1)** or KA+TAT-Cx43_266–283_ **(C1)**. The lesioned area was delimited by a dashed red line and quantified along the rostrocaudal axis, 7 days after KA injection **(A2)**, KA+TAT **(B2)** or KA+TAT-Cx43_266–283_ **(C2)**. The areas of at least 50 sections per animal were quantified. The results from three independent experiments were plotted on each graph. **(D)** Quantification of neuronal death volume (means ± SEM *n* = 3). *p < 0.05 ANOVA post-test (Holm-Sidak method for multiple comparisons). Bar, 250 μm.

Indeed, when the neuronal death area was estimated along the rostrocaudal axis (Figure 2), there was a strong reduction in neuronal death in animals injected with KA+TAT-Cx43_266–283_ (Figure 2C1) compared to KA (Figure 2A1) or KA+TAT (Figure 2B1). Quantification of neuronal death areas along the rostrocaudal axis showed a reduction in the height and length of the curves in mice treated with KA+TAT-Cx43_266–283_ (Figure 2C2) compared to KA (Figure 2A2) or KA+TAT (Figure 2B2). The area under these curves was calculated, using Cavalieri’s principle (described in “Materials and Methods” section) as an estimation of the volume of these lesions (Figure 2D). These results confirmed that TAT-Cx43_266–283_ strongly reduced the extent of neuronal death promoted by KA injection (Figure 2D).

### Astrocytes Are Involved in the Neuroprotective Effect of TAT-Cx43_266–283_

**Figure 3:**
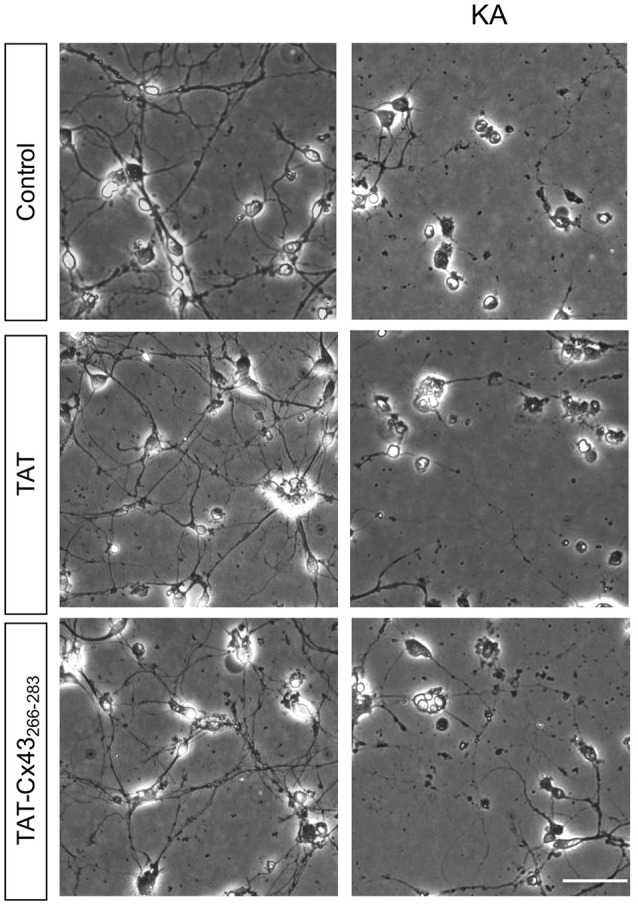
Effect of TAT-Cx43_266–283_ on neuronal death promoted by KA in cultured neurons. Representative phase-contrast photomicrographs showing that 100 μM KA induced neurotoxicity in cultured neurons in the presence or absence of 50 μM TAT or TAT-Cx43_266–283_ for 72 h. Bar, 20 μm.

**Figure 4:**
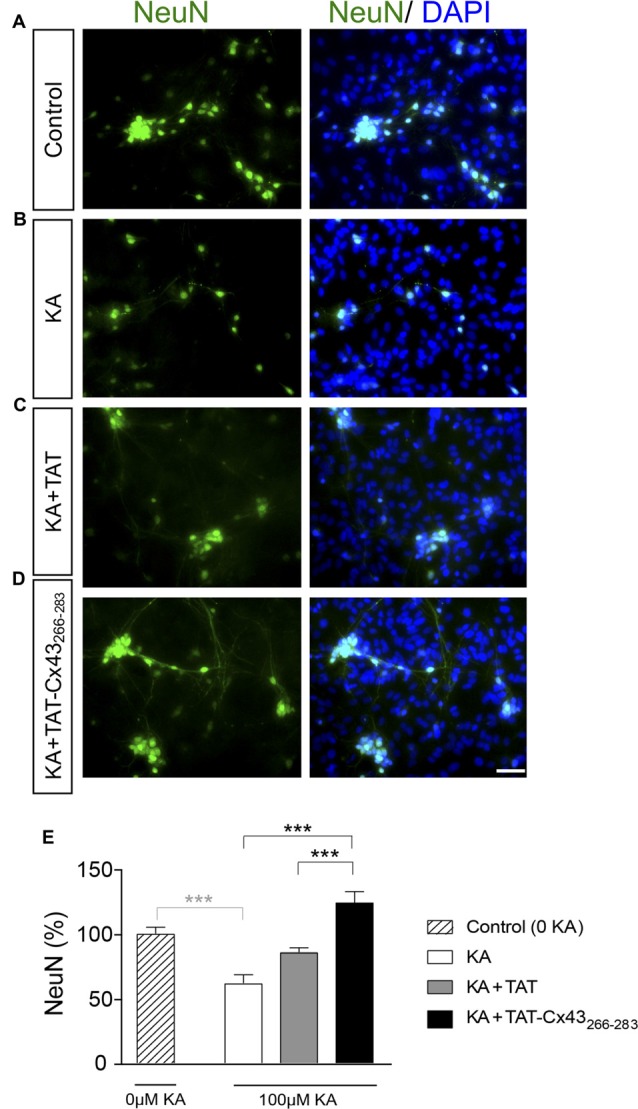
Effect of TAT-Cx43_266–283_ on neuronal death promoted by KA in neuron-astrocyte co-cultures. Representative photomicrographs of NeuN immunocytochemistry (green) and 4′,6′-diamidino-2-phenylindole (DAPI) nuclear staining (blue) in control neuron-astrocyte co-cultures **(A)** or co-cultures treated with 100 μM KA **(B)**, 100 μM KA + 50 μM TAT **(C)** or 100 μM KA + 50 μM TAT-Cx43_266–283_ **(D)** for 8 h. **(E)** Bar chart showing the ratio of NeuN-positive cells against the total number of cells stained with DAPI per field. The results are the means ± SEM (*n* = 4) and are expressed as percentages of the control (0KA). ***p < 0.001 ANOVA (post-test Holm-Sidak). Bar, 20 μm.

In order to gain insight into the neuroprotective mechanism of TAT-Cx43_266–283_ we used cell cultures to address the contribution of each type of cell to this process. First, the effect of KA and TAT-Cx43_266–283_ in neurons from primary cultures was analyzed. Surprisingly, TAT-Cx43_266–283_ did not protect against neuronal death promoted by KA in these cultures (Figure 3), suggesting the participation of a glial cells partnership in the neuroprotective effect of TAT-Cx43_266–283_. Indeed, when neurons were grown on top of astrocyte cultures, TAT-Cx43_266–283_ prevented neuronal death promoted by KA (Figure 4). The number of NeuN-positive cells decreased in neuron-astrocyte co-cultures in the presence of KA (Figure 4A vs. Figure 4B). However, when KA was added together with TAT-Cx43_266–283_ (Figure 4D) the number of NeuN-positive cells was not reduced when compared with the control (Figure 4A). Quantification of this data (Figure 4E) confirmed the preventive effect of TAT-Cx43_266–283_ on the reduction of NeuN-positive cells promoted by KA treatment in neuron-astrocyte co-cultures. It should be mentioned that TAT-Cx43_266–283_ did not affect NeuN staining in neuron-astrocyte co-cultures in the absence of KA (Supplementary Figure S1). In agreement with previous studies (Meloni et al., 2014), TAT also showed a slight neuroprotective effect, although it was lower to that found with TAT-Cx43_266–283_, suggesting that the region 266–283 in Cx43 has a neuroprotective effect.

**Figure 5:**
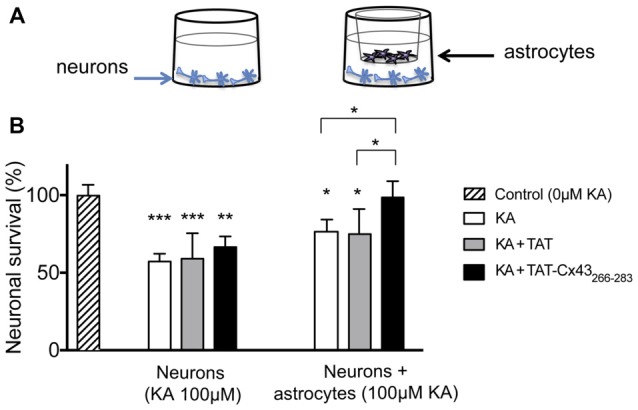
Effect of TAT-Cx43_266–283_ on neuronal death promoted by KA in non-contact neuron-astrocyte co-cultures. **(A)** Schematic of the non-contact neuron-astrocyte co-culture system used. **(B)** Neurons were plated alone or in co-culture with astrocytes. After incubation in the absence (control) or presence of 100 μM KA, 100 μM of KA + 50 μM TAT or 100 μM KA + 50 μM TAT-Cx43_266–283_ for 72 h, the neuronal viability was analyzed by MTT. The results are expressed as the percentages of the absorbance found in the control and are the mean ± SEM (*n* = 3). ***p < 0.001, **p < 0.01, *p < 0.05; ANOVA (post-test Holm-Sidak).

To address whether a change in the production of diffusible molecule was involved in the neuroprotective effect, non-contact neuron-astrocyte co-cultures were used. Astrocytes cultured within inserts were placed into wells containing neuronal cultures, as illustrated in Figure 5A. After different treatments, astrocyte inserts were removed and neuronal viability was estimated by MTT viability assays as described in “Materials and Methods” section. In parallel, neurons were subjected to the same treatments in the absence of astrocyte inserts. Consistent with the images shown in Figure 3, in the absence of glial cells TAT-Cx43_266–283_ did not significantly modify neuronal death promoted by KA (Figure 5B). However, in the presence of inserts containing astrocytes, TAT-Cx43_266–283_ prevented neuronal death promoted by KA (Figure 5B), suggesting that soluble factors released by astrocytes are contributing to this effect.

### Astrocyte Hemichannels Contribute to KA-Induced Neurotoxicity

**Figure 6:**
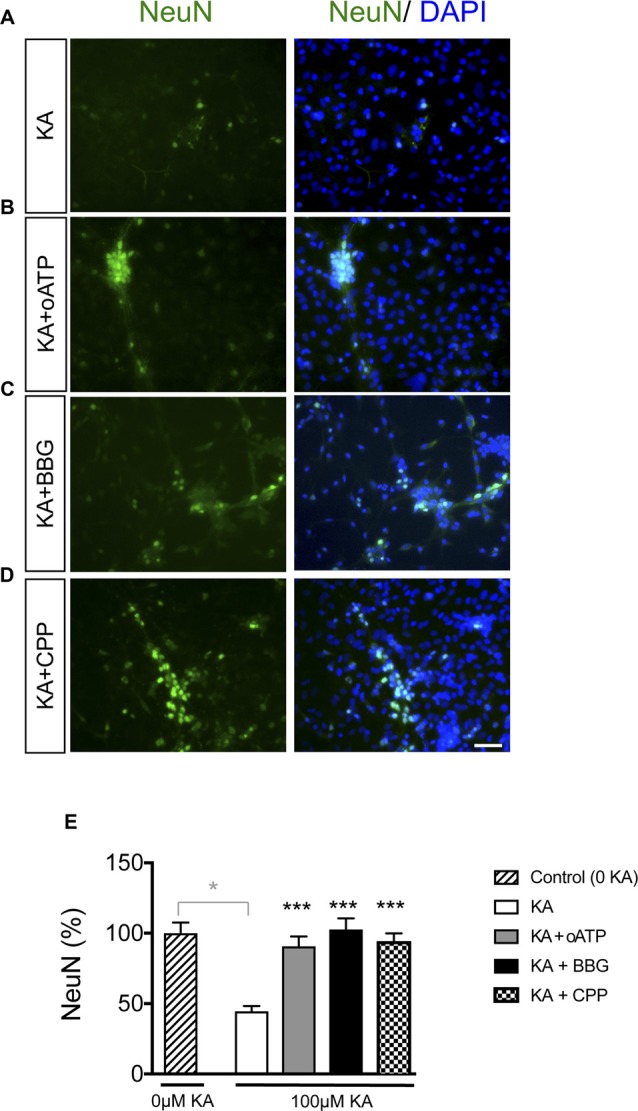
Effect of glutamate and ATP on neuronal death promoted by KA in neuron-astrocyte co-cultures. Representative photomicrographs of NeuN immunocytochemistry (green) and DAPI nuclear staining (blue) in neuron-astrocyte co-cultures treated with 100 μM KA **(A)**, 100 μM KA + 200 μM oATP (P2X receptor blocker; **B**), 100 μM KA + 100 μM BrilliantBlue G (BBG; P2X7 receptor blocker; **C**) or 100 μM KA + 20 μM CPP (NMDA receptor blocker; **D**) for 8 h. **(E)** Bar chart showing the ratio of NeuN-positive cells against the total number of cells stained with DAPI per field. The results are the means ± SEM (*n* = 3) and are expressed as percentages of the control (0KA). *p < 0.05 vs. control (0 KA), ***p < 0.001 vs. KA; ANOVA (post-test Holm-Sidak). Bar, 20 μm.

It has been extensively documented that astrocyte hemichannels when activated release several factors, such as glutamate and ATP that cause neuronal death (Orellana et al., 2011a). Therefore, we explored the participation of these channels in KA-induced neurotoxicity_._ Adding 200 μM oATP (P2X receptor blocker), 100 μM BBG (P2X7 receptor blocker) or 20 μM CPP (NMDA receptor blocker) during KA incubation prevented the reduction in NeuN-positive cells caused by KA in neuron-astrocyte co-cultures (Figure 6). Together these data confirmed that inhibitors of ATP- and NMDA-receptors prevented neuronal death in co-cultures treated with KA, suggesting the participation of glutamate and ATP release associated with astrocyte hemichannel activity in this neurotoxic process.

### TAT-Cx43_266–283_ Inhibits Astrocyte Hemichannel Activity Through c-Src

**Figure 7:**
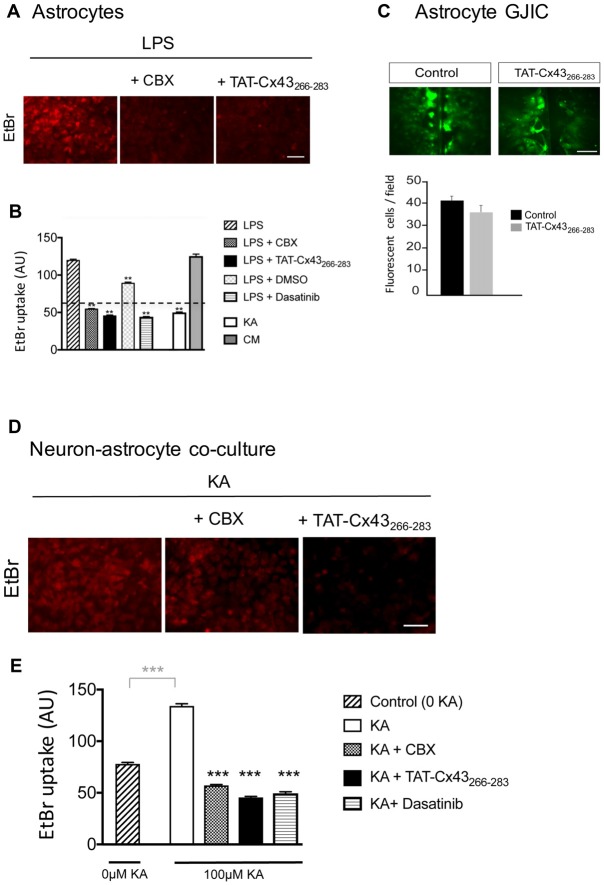
Effect of TAT-Cx43_266–283_ on astrocyte hemichannel activity and gap junctional intercellular communication (GJIC) in astrocyte cultures and in neuron-astrocyte co-cultures. **(A,B)** Astrocyte cultures were incubated with 10 μg/ml lipopolysaccharide (LPS) for 24 h or with 100 μM KA or conditioned medium from KA-treated neuron-astrocyte co-cultures (CM) for 8 h. Then, 5 μM ethidium bromide (EtBr) was added for 10 min. Two-hundred micromolar Carbenoxolone (CBX), 50 μM TAT-Cx43_266–283_, 1 μM dasatinib and 1 μl/ml dimethyl sulfoxide were applied in the presence of LPS, 15 min before and during EtBr uptake. **(A)** Representative photomicrographs showing EtBr uptake in astrocytes after LPS treatment and the inhibition promoted by CBX and TAT-Cx43_266–283_. **(B)** Quantification of EtBr uptake intensity in arbitrary units (AU). Dotted line represents the basal level of EtBr fluorescence found in untreated astrocytes. The results are the means ± SEM (*n* = 3; at least 300 cells were counted per condition). **p < 0.01, ANOVA (post-test Holm-Sidak). **(C)** Astrocyte cultures were incubated in the absence or presence of 50 μM TAT-Cx43_266–283_ 24 h before and during the scrape-loading experiment. Photomicrographs obtained after Lucifer yellow (LY) scrape loading and quantification of the number of fluorescent cells per field. The results are the means ± SEM (*n* = 3). **(D,E)** Neuron-astrocyte co-cultures were incubated with 100 μM KA for 8 h and then 5 μM EtBr was added for 10 min. Two-hundred micromolar CBX, 50 μM TAT-Cx43_266–283_ or 1 μM dasatinib were applied in the presence of KA, 15 min before and during EtBr uptake. **(D)** Representative photomicrographs showing EtBr uptake in neuron-astrocyte co-cultures after KA treatment and the inhibition promoted by CBX and TAT-Cx43_266–283_. **(E)** Quantification of EtBr uptake intensity in AU. The results are the means ± SEM (*n* = 3; at least 300 cells were counted per condition). ***p < 0.001 ANOVA (post-test Holm-Sidak). Bar, 50 μm.

Because astrocyte hemichannels appeared to be involved in KA-mediated neuronal death (Figure 6), we hypothesized that TAT-Cx43_266–283_ could prevent hemichannel activation with the subsequent neuroprotective effect. To address this point, first we analyzed the effect of TAT-Cx43_266–283_ on glial hemichannel activity by EtBr uptake (Figure 7). Under resting conditions, glial hemichannel activity is negligible (Figure 7B, dotted line), therefore astrocytes were exposed to LPS for 24 h to increase hemichannel activity (Retamal et al., 2007; Orellana et al., 2011a). As shown in Figure 7A, LPS-treated astrocytes showed a strong uptake of the hemichannel permeable tracer. This effect was suppressed by CBX, a hemichannel inhibitor, applied in the presence of LPS, 15 min before and during the EtBr uptake (Figures 7A,B), demonstrating that EtBr uptake occurred through hemichannels. Interestingly, TAT-Cx43_266–283_ applied in the same way, i.e., in the presence of LPS, 15 min before and during the EtBr uptake, strongly reduced the activation of hemichannels promoted by LPS (Figures 7A,B). Since TAT-Cx43_266–283_ is an inhibitor of c-Src (Gangoso et al., 2014), which is an important mediator of neuroinflammation (Paul et al., 2001; Tai et al., 2013), we investigated the effect of dasatinib, a potent inhibitor of Src, on hemichannel activity. Dasatinib inhibited astrocyte hemichannel activity promoted by LPS (Figure 7B), revealing a role of c-Src in the regulation of hemichannel activity. Furthermore, the results obtained with dasatinib were very similar to those obtained with TAT-Cx43_266–283_ suggesting that TAT-Cx43_266–283_, by inhibiting c-Src, reduced hemichannel activity in astrocytes. We also analyzed the effect of TAT-Cx43_266–283_ on the communication through gap junctions in astrocytes (Figure 7C). The scrape loading assay showed that TAT-Cx43_266–283_ did not significantly modify gap junction intercellular communication between astrocytes.

To test the involvement of hemichannel activity on the neuroprotective effect of TAT-Cx43_266–283_ in KA-induced neurotoxicity, EtBr uptake was analyzed in neuron-astrocyte co-cultures treated with KA. Figures 7D,E show that KA strongly increased hemichannel activity in these co-cultures. Interestingly, TAT-Cx43_266–283_ applied in the presence of KA strongly inhibited hemichannel activity (Figures 7D,E). In fact, the inhibition promoted by TAT-Cx43_266–283_ was similar to that promoted by CBX, a hemichannel inhibitor, or by dasatinib, a c-Src inhibitor (Figures 7D,E). These data suggest that TAT-Cx43_266–283_ by inhibiting c-Src activity prevented hemichannel activity promoted by KA in neuron-astrocyte co-culture.

**Figure 8:**
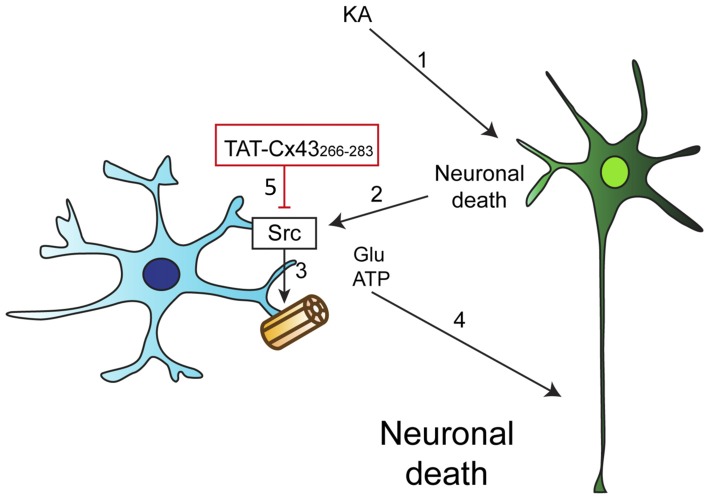
Proposed mechanism of TAT-Cx43_266–283_ neuroprotective effect. (1) KA induces neuronal death by excitotoxicity. (2) This injury promotes the release of proinflammatory molecules that cause the activation of c-Src in astrocytes. (3) c-Src, among other effects, can activate astrocyte hemichannels, causing the release of some factors, such as ATP and glutamate. (4) ATP and glutamate increase the extent of neuronal death. (5) TAT-Cx43_266–283_ inhibits c-Src activity and consequently glial hemichannel activity with the subsequent neuroprotective effect.

We then investigated whether KA, *per se*, affected astrocyte hemichannel activity. Figure 7B shows that KA did not activate hemichannels in astrocytes. However, when astrocytes were exposed to conditioned medium from KA-treated neuron-astrocyte co-cultures, the activity of astrocyte hemichannels strongly increased (Figure 7B). These results confirm that some factors released by damaged neurons, but not KA, activate astrocyte hemichannels. Altogether, these data suggest that TAT-Cx43_266–283_, by inhibiting c-Src, prevents the activation of hemichannels caused by factors released by KA-damaged neurons (Figure 8).

## Discussion

Our previous studies reported that a cortical injection of KA promoted a transient increase in c-Src activity in the region surrounding the neuronal lesion (Gangoso et al., 2012). c-Src is an important mediator in several signaling pathways related to neuroinflammation (Paul et al., 2001; Tai et al., 2013). In this study, we revealed that TAT-Cx43_266–283_, a Cx43 mimetic peptide that inhibits c-Src activity (Gangoso et al., 2014; González-Sánchez et al., 2016; Jaraíz-Rodríguez et al., 2017), reduces the extent of neuronal death promoted by KA.

Intriguingly, we show here that the neuroprotective effect of TAT-Cx43_266–283_ in KA-injured neurons requires the presence of astrocytes. Clearly, the initial neuronal injury promoted by KA is due to excitotoxicity. As a potent agonist for the AMPA/kainate class of glutamate receptor, KA increases the production of reactive oxygen species, mitochondrial dysfunction and apoptosis in neurons (Wang et al., 2005). Similarly to other neuronal injuries, this primary tissue damage induces subsequent neurotoxic factor release, resulting in the activation of microglia cells and astrocytes that, although initially exert a neuroprotective effect, in the long term contributes to the delayed neuronal death (Hong et al., 2010; Zhang and Zhu, 2011). Although further research is required, it could be proposed that TAT-Cx43_266–283_, by inhibiting c-Src, interferes with the neuroinflammatory response evoked by activated astrocytes after KA-induced neuronal death. In agreement with this, KA promotes a transient increase in c-Src activity in activated astrocytes surrounding KA-injured neurons (Gangoso et al., 2012). Moreover, the present study shows that the inhibitor of c-Src, TAT-Cx43_266–283_, reduces the activation of astrocytes that follows KA lesion.

Astrocytes are known to represent the brain cell population that expresses the higher among of connexins forming hemichannels. Moreover, increasing evidence suggests that inflammation-induced astrocyte hemichannel activation plays a critical role in neuronal death (Bennett et al., 2012; Ishii et al., 2013; Yi et al., 2016; Belousov et al., 2017; Gajardo-Gómez et al., 2017). Thus, astrocyte hemichannels in neuron-astrocyte co-cultures participate in NMDA-induced neurotoxicity (Froger et al., 2010). The activation of astrocyte hemichannels releases ATP and glutamate that contribute to neuronal death (Orellana et al., 2011a; Bennett et al., 2012). Although our results did not demonstrate that the release of ATP and glutamate occurs directly from hemichannels, because it was not under the scope of this study, they showed a neurotoxic effect of ATP and glutamate that takes place when astrocyte hemichannels are opened. Indeed, according to our results, ATP and glutamate participate in neuronal death promoted by KA in neuron-astrocyte co-culture, suggesting the participation of astrocyte hemichannels in the neurotoxic effect caused by KA. In agreement with this, the results presented in this study showed that some factors released by damaged neurons promoted the activation of astrocyte hemichannels and neuronal death. Interestingly, this effect was impaired by TAT-Cx43_266–283_, which inhibited astrocyte hemichannel activity. The astrocyte hemichannel activation appears to be mediated by c-Src, first because TAT-Cx43_266–283_ has been reported to be an important inhibitor of c-Src (Gangoso et al., 2014; González-Sánchez et al., 2016; Jaraíz-Rodríguez et al., 2017) and also because other inhibitors of c-Src, such as dasatinib, inhibited astrocyte hemichannel activity.

Neuronal injury causes the release of several pro-inflammatory cytokines such as TNF-alpha, IL-1or IL-6 (Feuerstein et al., 1994) that can activate c-Src (Yu et al., 2007; Byeon et al., 2012; Liu et al., 2014; Song et al., 2016) and astrocyte hemichannels (Froger et al., 2010). In fact, c-Src activation and glial hemichannel activity are common events in several CNS diseases, suggesting a link between them. Thus, the deleterious contribution of c-Src activity to neurodegeneration have been extensively documented in models of Parkinson’s disease (Tai et al., 2013; Wang et al., 2016), Alzheimer’s disease (Dhawan and Combs, 2012; Kaufman et al., 2015) and stroke (Paul et al., 2001). Similarly, astrocytes open their Cx43 hemichannels in Parkinson’s disease (Rufer et al., 1996; Kawasaki et al., 2009) Alzheimer’s disease (Orellana et al., 2011b; Takeuchi et al., 2011; Yi et al., 2016) and ischemia (Contreras et al., 2002; Retamal et al., 2006). We found that c-Src inhibitors reduce astrocyte hemichannel activity promoted by LPS or by damaged neurons, suggesting that c-Src activity increases astrocyte hemichannel activity. To our knowledge, there is no evidence indicating that c-Src directly opens Cx43 hemichannels. c-Src binds to the SH3 domain binding motif of Cx43 and then phosphorylates tyrosines 265 and 247. As a consequence, gap junctional intercellular communication (GJIC) is reduced, and Cx43 turnover is initiated (Kanemitsu et al., 1997; Giepmans et al., 2001; Sorgen et al., 2004). Although other possibilities cannot be ruled out, these changes in Cx43 conformation and/or location might be responsible for the increase in hemichannel activity. Consistent with this, pioneer studies on Cx43 mimetic peptides showed that the sequence 271–287 injected into Xenopus oocytes prevented the inhibition of Cx43 gap junction channels promoted by intracellular acidification (Calero et al., 1998). Intracellular acidification increases c-Src activity (Yamaji et al., 1997) and reduces gap junctional communication (Li et al., 2005), suggesting a link between both processes (Lau, 2005). Therefore, we speculated that the sequence 271–287, similarly to TAT-Cx43_266–283_, could inhibit c-Src activity by recruiting PTEN and CSK (González-Sánchez et al., 2016; Jaraíz-Rodríguez et al., 2017) interfering with the effects of c-Src on Cx43-channel properties.

Taken together, these results allow us to propose that astrocyte hemichannel activity is increased after KA-neuronal injury and that TAT-Cx43_266–283_, by inhibiting c-Src, among other effects reduces astrocyte hemichannel activity with the subsequent neuroprotective effect (Figure 8). Furthermore, it should be mentioned that TAT-peptides can be delivered *in vivo* in mice, and display penetration into the brain parenchyma (Abudara et al., 2014; Stalmans et al., 2015), which increase the interest of TAT-Cx43_266–283_ for the development of new therapies to reduce excitotoxic-mediated neurodegeneration. Additionally, the results obtained with this Cx43 mimetic peptide may provide some clues about the role played by the changes in Cx43 expression found in astrocytes under pathological conditions (Giaume et al., 2010). In this sense, in addition to affect Cx43 channel functions, changes in Cx43 expression can modulate c-Src activity. Thus, down-regulation of Cx43 in astrocytes increases c-Src activity (Gangoso et al., 2012; Valle-Casuso et al., 2012). Conversely, if TAT-Cx43_266–283_ mimics the effect of the up-regulation of Cx43, the increase in astrocyte Cx43 expression would reduce c-Src activity with the subsequent reduction in hemichannel activity and neuroprotective effect. Finally, this study opens the interesting possibility that glial hemichannels could be involved in the neuroinflammatory response mediated by c-Src in major CNS diseases like Parkinson’s disease, Alzheimer’s disease or stroke (Paul et al., 2001; Dhawan and Combs, 2012; Tai et al., 2013; Kaufman et al., 2015; Wang et al., 2016).

## Author Contributions

EG, RT, MJ-R and MD-P contributed to the experimental development, data acquisition, analysis and interpretation and drafting the article. PE contributed to the experimental development, data acquisition and analysis. AK and JMM contributed to the experimental design and data interpretation. CG conceived and designed the experiments, designed and processed documentation for bioethics committee approval from Collège de France (France), supervised the experimental development and analysis, interpreted the data and contributed to drafting the article. AT conceived and designed the experiments, designed and processed documentation for bioethics committee approval from the University of Salamanca and Junta de Castilla y León (Spain), supervised the experimental development and analysis, interpreted the data and drafted the article. All authors revised the article for important intellectual content and approved of the final version for publication.

## Conflict of Interest Statement

The authors declare that the research was conducted in the absence of any commercial or financial relationships that could be construed as a potential conflict of interest. The handling Editor declared a past co-authorship with one of the authors, CG.
