# Supplementary figures and images for: A c-Src Inhibitor Peptide Based on Connexin43 Exerts Neuroprotective Effects through the Inhibition of Glial Hemichannel Activity
Source: Front Mol Neurosci. 2017 Dec 15;10:418. doi: 10.3389/fnmol.2017.00418 (PMC5737028; doi:10.3389/fnmol.2017.00418)

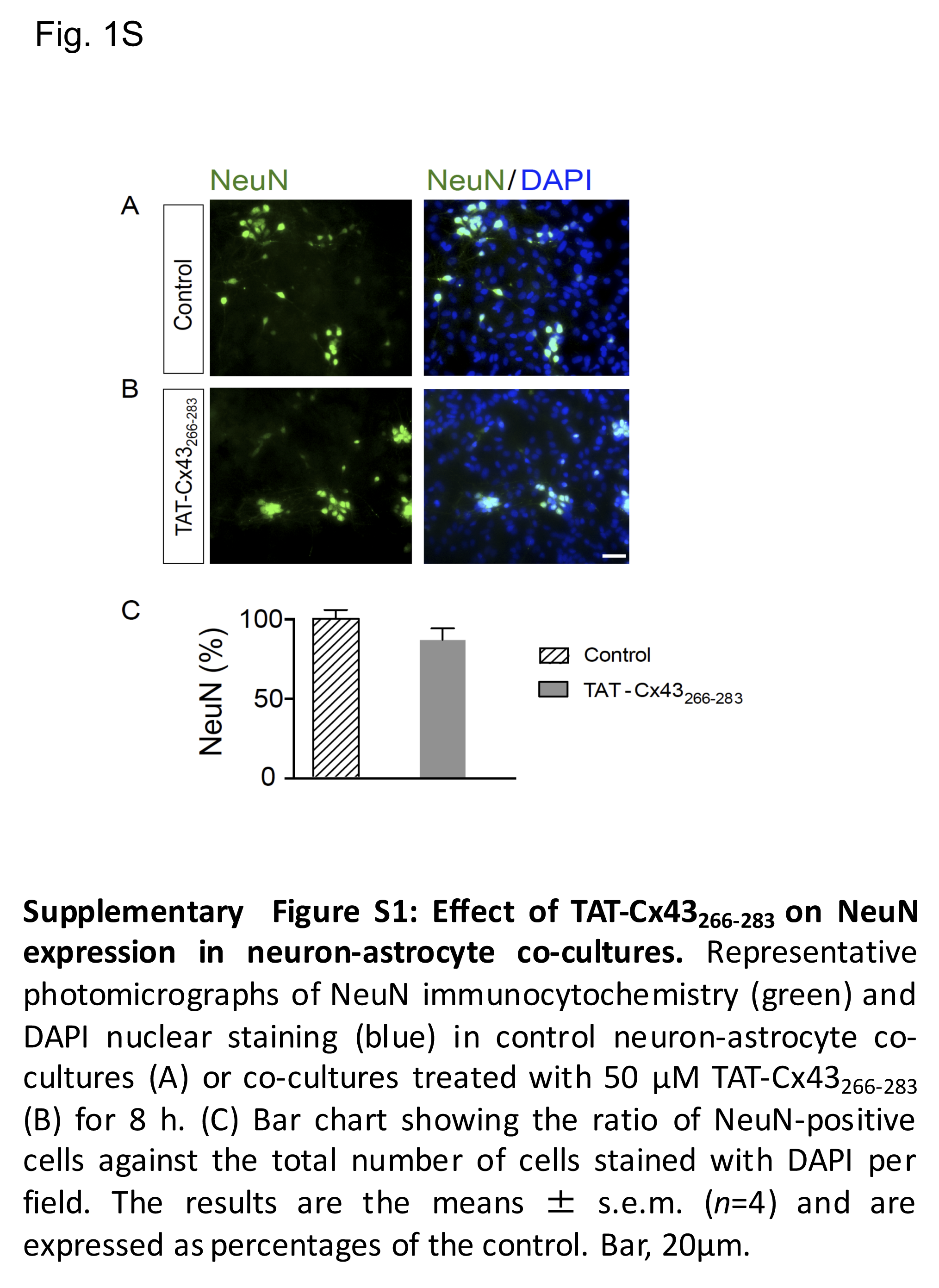

Supplement: Supplementary file 1 [file Image_1.tif]
